# Quantifying anthropogenic wolf mortality in relation to hunting regulations and landscape attributes across North America

**DOI:** 10.1002/ece3.8875

**Published:** 2022-05-20

**Authors:** Jacob E. Hill, Hailey M. Boone, Mariela G. Gantchoff, Todd M. Kautz, Kenneth F. Kellner, Elizabeth K. Orning, Jamshid Parchizadeh, Tyler R. Petroelje, Nathaniel H. Wehr, Shannon P. Finnegan, Nicholas L. Fowler, Ashley L. Lutto, Sarah L. Schooler, Merijn van den Bosch, Alejandra Zubiria Perez, Jerrold L. Belant

**Affiliations:** ^1^ Global Wildlife Conservation Center State University of New York College of Environmental Science and Forestry Syracuse New York USA; ^2^ Savannah River Ecology Laboratory University of Georgia Aiken South Carolina USA; ^3^ U.S. Geological Survey Fort Collins Science Center Fort Collins Colorado USA

**Keywords:** *Canis lupus*, carnivore, cause‐specific mortality, meta‐analysis, telemetry

## Abstract

Understanding the types and magnitude of human‐caused mortality is essential for maintaining viable large carnivore populations. We used a database of cause‐specific mortality to examine how hunting regulations and landscape configurations influenced human‐caused mortality of North American gray wolves (*Canis lupus*). Our dataset included 21 studies that monitored the fates of 3564 wolves and reported 1442 mortalities. Human‐caused mortality accounted for 61% of mortality overall, with 23% due to illegal harvest, 16% due to legal harvest, and 12% the result of management removal. The overall proportion of anthropogenic wolf mortality was lowest in areas with an open hunting season compared to areas with a closed hunting season or mixed hunting regulations, suggesting that harvest mortality was neither fully additive nor compensatory. Proportion of mortality from management removal was reduced in areas with an open hunting season, suggesting that legal harvest may reduce human‐wolf conflicts or alternatively that areas with legal harvest have less potential for management removals (e.g., less livestock depredation). Proportion of natural habitat was negatively correlated with the proportion of anthropogenic and illegal harvest mortality. Additionally, the proportion of mortality due to illegal harvest increased with greater natural habitat fragmentation. The observed association between large patches of natural habitat and reductions in several sources of anthropogenic wolf mortality reiterate the importance of habitat preservation to maintain wolf populations. Furthermore, effective management of wolf populations via implementation of harvest may reduce conflict with humans. Effective wolf conservation will depend on holistic strategies that integrate ecological and socioeconomic factors to facilitate their long‐term coexistence with humans.

## Graphical abstract

Wolf mortality from management removal was reduced when harvest was legal, suggesting that legal harvest may reduce human–wolf conflicts. Anthropogenic mortality of wolves was greatest in landscapes with less natural habitat.

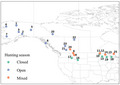

## 1 INTRODUCTION

Widespread persecution and habitat loss have precipitated the decline of many North American carnivore populations (e.g., Laliberte & Ripple, 2004). However, recovery for some species has been achieved through legal protections, habitat preservation, and increasing tolerance for wild predators (George & Crooks, 2006; Gompper et al., 2015; Linnell et al., 2001). Integral to species demography, mortality plays a substantial role in the growth and persistence of carnivore populations (Krebs et al., 2004; Moss et al., 2016). Human‐induced mortality in particular can influence species by affecting individuals from different demographic groups (e.g., individuals with greater reproductive value) compared with natural mortality sources (Gunson et al., 2003; Wright et al., 2006). Consequently, shifts in mortality from natural to human‐induced causes could produce selection of behavioral, demographic, or physical attributes with subsequent effects to population dynamics (Ciuti et al., 2012; Coltman et al., 2001, 2003).

Common sources of anthropogenic mortality for carnivores in North America include harvest, vehicle mortality, and management removal (i.e., killed in accordance with a depredation permit or in defense of life or property) (Hill et al., 2019b). However, the proportion of human‐caused mortality across carnivore populations varies because anthropogenic mortality is influenced by factors such as landscape attributes and densities of conspecifics (Hill et al., 2020; Murray et al., 2010). For example, anthropogenic mortality of large mammals increases with greater levels of human influence on the landscape including greater human population density, changes in land use, and road networks (Hill et al., 2020; Wynn‐Grant et al., 2018). Although some animals move less in areas with greater human disturbance, habitat fragmentation can lead to increases in anthropogenic mortality when extensive movements by individual animals are necessary to meet resource requirements (Hussain et al., 2007; Tucker et al., 2018). Carnivores may experience higher rates of harvest near roads due to ease of human access (Nielsen et al., 2004). Additionally, carnivores may face elevated anthropogenic mortality through management removal in land cover types where conflict with humans is more likely to occur (Wynn‐Grant et al., 2018).

In addition to landscape characteristics, hunting regulations directly influence the proportion of mortality attributable to anthropogenic causes, especially for species subject to high levels of harvest (Hill et al., 2019b). Globally, the proportion of mammal mortality from anthropogenic sources increases when legal hunting is permitted (Hill et al., 2019b). For some carnivore species, total anthropogenic mortality can increase by an order of magnitude in areas with an open hunting season (Gantchoff et al., 2020). Conversely, killing of carnivores by management removal may decrease when a hunting season is instituted (Olson et al., 2015; Raithel et al., 2017). The role of harvest regulations on anthropogenic mortality can be especially complex for wide‐ranging carnivores inhabiting expansive areas that constitute a matrix of varying hunting regulations (Obbard et al., 2017).

For reestablishing populations, the length of time the species has been in the area may also influence mortality patterns. For example, there may initially be high levels of conflict with humans that abate over time as mitigation strategies are implemented (Strand et al., 2019). Additionally, people may become more tolerant of reestablishing species as they become more accustomed to them (Zimmermann et al., 2001). Consequently, the reestablishment status of a population may influence cause‐specific mortality along with harvest regulations and landscape attributes. Understanding how these factors interact to affect cause‐specific mortality is important for the development of management plans and may help to identify factors that could limit or improve recovery of carnivore populations.

Gray wolf (*Canis lupus*) populations have recovered in portions of their historic range in North America due to successful reintroduction efforts and natural expansion after severe population declines in the mid‐20th century (United States Fish & Wildlife Service, 2014). Wolves were nearly extirpated from the contiguous United States and adjacent portions of Canada by the 1930s, primarily to protect livestock producers from the threat of depredation (Stone et al., 2017). Over the past half century, wolves in North America have had a dynamic legal status and been subject to fluctuating harvest regulations. These have included indiscriminate killing, recreational hunting seasons, and permits to kill wolves that cause property damage (i.e., livestock damage) (Olson et al., 2015). Their expansive range results in widespread variability in mortality sources, including both killing as the result of human‐wildlife conflict and harvest for consumption by indigenous communities (Fritts et al., 2003).

Assessing vertebrate cause‐specific mortality with telemetry can produce less biased estimates than other techniques such as opportunistic encounters of dead individuals (Naef‐Daenzer et al., 2017). We used a database of telemetry‐based cause‐specific mortality to test the hypotheses that landscape attributes, reestablishment status, and harvest regulations influenced cause‐specific mortality of gray wolves from anthropogenic sources (i.e., legal harvest, illegal harvest, management removal, and overall anthropogenic mortality). Specifically, we predicted the proportion of mortalities due to anthropogenic causes would increase in areas with an open wolf hunting season. We predicted the proportion of mortalities due to illegal harvest would increase when legal hunting was prohibited, and that management removal would decrease when wolf hunting was allowed through a hunting season. Additionally, we predicted the proportion of mortalities due to anthropogenic causes would increase when landscapes were fragmented (owing to increased wolf movements resulting in greater risk of encountering humans and human‐wolf conflicts), when road densities were greater (resulting from increased human access), and at lower proportions of natural habitat (due to the increased presence of humans). We also predicted an increase in management removal in areas with lower proportions of natural habitat and increased road density due to increased contact with humans. Lastly, we predicted that reestablished populations would experience higher levels of mortality from management removal and overall anthropogenic causes.

## 2 MATERIALS AND METHODS

### 2.1 Study area

Historically, the distribution of wolves in North America spanned throughout coastal Greenland, Canada, western and central United States, and central Mexico (Nowak, 2002). Following years of intentional wolf removal (Fritts et al., 2003), the current distribution of wolves in North America is limited to the western Great Lakes and northern Rocky Mountain regions of the contiguous United States, much of Canada and Alaska, and coastal Greenland with a few remnant populations of wolves found elsewhere (Boitani et al., 2018). Our study area included the present range (i.e. 2022) of North American gray wolves.

### 2.2 Data extraction

We retrieved initial data for our meta‐analysis from CauseSpec, a database of global terrestrial vertebrate cause‐specific mortality studies (Hill et al., 2019a). Studies in the database monitored animals using telemetry, and mortalities were investigated when possible to determine cause of death. We selected papers from the database including gray wolves. We did not include studies of eastern wolves (*C. l. lycaon*) due to uncertainty regarding their status as a separate species (Heppenheimer et al., 2018; Nowak, 2002), or Mexican gray wolves (*Canis lupus baileyi*) due to this population's isolated location relative to the remainder of *C. lupus* in North America, and the unique management conditions influencing them (Povilitis et al., 2006). We replicated methods described by Hill et al. (2019a), adding “wolf” and “canis lupus” to search terms for additional publications, technical reports, or theses published after CauseSpec. We removed studies that duplicated data already represented or failed to report cause‐specific mortality of radio‐collared individuals as unique values separate from other mortality reports (Hill et al., 2019a). Studies of *C*. *lupus* outside of North America were excluded due to low sample size (*n* = 7 studies, 147 mortalities of known cause). We classified sources of mortality as anthropogenic (e.g., harvest and vehicle collisions), natural (e.g., disease and starvation), or unknown.

We first categorized the study sites in relation to hunting regulations as having an open hunting season (i.e., during the study period wolves were delisted and one or more hunting seasons were allowed), or closed hunting season (i.e., hunting of wolves was prohibited throughout the study period). If a study included multiple distinct study sites with different wolf hunting regulations in each area, we considered the study sites as separate data points. If a study included multiple time periods with distinct hunting regulations, we separated the study into unique study sites by time period. However, we also created a third category called “Mixed hunting regulations,” which included studies where (a) a hunting season was implemented during the course of the study, but mortalities before and after the regulatory change were not reported separately, and (b) studies for which the study area was large enough to include areas open and closed to hunting. For each study, we also recorded if the wolf population was permanent or had reestablished (through natural recolonization or reintroduction). We used study area maps from each publication to construct a polygon representing the study site. For each polygon, we extracted covariates related to human disturbance including road density and land cover fragmentation correlates. We estimated mean road density (m/km^2^) using the global roads inventory project database (Meijer et al., 2018). We masked rasterized land covers using ArcGIS 10.3.1 (ESRI, 2015) from the North American Land Cover dataset (NALCD, 30‐m resolution) (Latifovic et al., 2017; Yang et al., 2018) to our study site polygons and reclassified those land covers into anthropogenic (i.e., urban and cropland) and natural (i.e., forest, shrubland, grassland, lichen, moss, snow and ice, open water, and wetland). We used the reclassified NALCD to calculate mean patch area of natural habitat, and proportion natural area for each study site polygon in R version 3.5.3 (R Core Team, 2020) using package landscapemetrics (Hesselbarth et al., 2019).

### 2.3 Data analysis

We considered four response variables in this analysis for each study datum: (a) proportion of mortalities caused by anthropogenic effects (all anthropogenic sources combined), (b) proportion of mortalities due to illegal harvest (harvested on a site where harvest was prohibited, outside the hunting season, or without requisite reporting required by local regulations), (c) proportion of mortalities due to legal harvest (limited only to studies with an open hunting season) and (d) proportion of mortalities due to management removal. An illegal harvest mortality could occur where an open hunting season existed if it occurred outside the months of the hunting season or was not properly reported to authorities. Each of these mortality response variables was calculated as the number dying from that cause divided by the total number of mortalities of known cause. We used this metric for analysis because we were unable to calculate mortality rates based on the information available in source publications. We used proportional mortality instead of absolute numbers of mortalities so that variation in mortality from various causes would not be a result of different numbers of wolves monitored across studies. We fit separate models for each response using logistic regression and assessed model predictive accuracy by calculating the area under the receiver operating characteristic curve (AUC; Swets, 1988). Covariates included in each model were hunting season status (open, closed, or mixed), proportion of natural habitat in the study area, mean patch size of natural habitat, whether the population was reestablished, and road density. All continuous covariates were standardized to have a mean of 0 and a standard deviation of 1 before analysis. We determined whether estimated coefficients differed from 0 using Wald tests, with ∝ < 0.05. For the model of proportion of mortalities due to illegal harvest, we encountered separation issues with the patch size covariate. This was likely due to five studies with much larger mean patch area than the rest of the studies, all of which had no illegal harvest mortality recorded. To address this, we fit the illegal harvest model using Firth's penalized‐likelihood logistic regression (Firth, 1993).

## 3 RESULTS

### 3.1 Data summary

**FIGURE 1:**
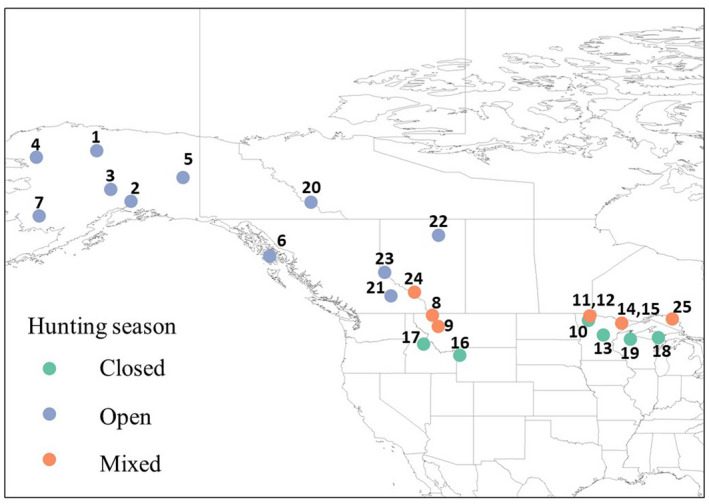
Locations of wolf mortality study sites (*n* = 25) from 21 studies in North America, 1968–2019. Numbers correspond to numbers in ID column in Table 1. The wolf hunting season status (see methods) for each study are identified by colored circles

**FIGURE 2:**
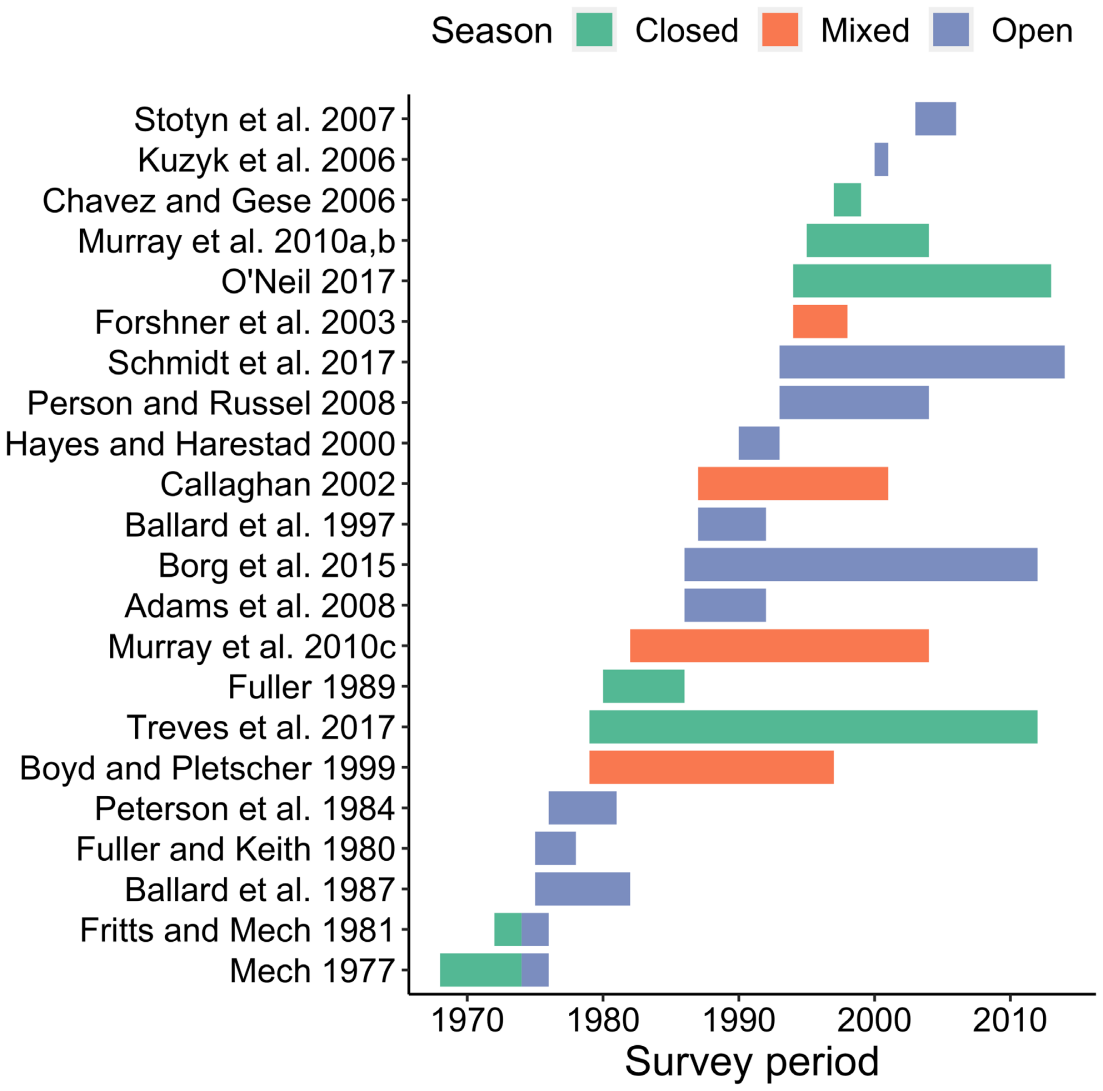
Timeline for 25 study sites (from 21 unique studies) used to model gray wolf (*Canis lupus*) mortality across North America, 1968–2019. In‐text citations on the y‐axis indicate which study was associated during a given period. Hunting season status (i.e., open, closed, and mixed) is denoted by colored bars, and data were retrieved from the CauseSpec database of global terrestrial vertebrate cause‐specific mortality studies and a review of wolf literature (see methods)

Using CauseSpec and additional recent literature, we initially identified 21 candidate studies of *C*. *lupus* to include in the analysis. All studies were in North America; 15 from the United States and 6 from Canada (Table 1, Figure 1). Of the 21, two studies (Fritts & Mech, 1981; Mech, 1977) comprised two time periods under different hunting season regimes, and we considered each as two separate study sites. One study (Murray et al., 2010) comprised three study areas that we considered separately. Thus, we had a total of 25 study sites in the final analyses of anthropogenic, illegal, and management mortality. For analysis of legal harvest mortality, we removed 8 study sites where hunting was prohibited, resulting in 17 study sites in that dataset. The range of studies spanned from 1968 to 2014 (Figure 2). Study sites were highly variable in size (mean + standard deviation: 37,625 + 62,120 km^2^) and ranged from 584 to 294,489 km^2^. Across all studies, 1,442 wolf mortalities were recorded with 329 (23%) due to illegal harvest, 225 (16%) due to legal harvest, and 174 (12%) due to management removal. Overall, 873 mortalities (61%) were human caused (Table 2).

**TABLE 1:** Causes of mortality from 3564 monitored gray wolves (*Canis lupus*) reported for 25 study sites (derived from 21 studies) across North America, 1968–2019

ID	Study area	Number monitored	Mortalities			Year	Citation
			Total	Illegal	Legal		
1	Central Brooks Range, Alaska, USA	50	20	0	9	1986–1992	Adams et al. (2008)
2	Southcentral, Alaska, USA	151	71	24	33	1975–1982	Ballard et al. (1987)
3	Southcentral, Alaska, USA	387	94	0	21	1986–2012	Borg et al. (2015)
4	Northwestern, Alaska, USA	85	52	0	36	1987–1992	Ballard et al. (1997)
5	Western, Alaska, USA	143	104	0	22	1993–2014	Schmidt et al. (2017)
6	Prince of Wales, Alaska, USA	55	39	16	18	1993–2004	Person and Russel (2008)
7	Kenai Peninsula, Alaska, USA	64	23	2	18	1976–1981	Peterson et al. (1984)
8	Northwestern, Montana, USA	58	31	0	17	1979–1999	Boyd and Pletscher (1999)
9	Northwestern, Montana, USA	193	131	39	20	1982–2004	Murray et al. (2010)
10	Northwestern, Minnesota, USA	16	9	4	0	1997–1999	Chavez and Gese (2006)
11	Northwestern, Minnesota, USA	23	7	4	2	1972–1974	Fritts and Mech (1981)
12	Northwestern, Minnesota, USA	37	9	0	4	1974–1976	Fritts and Mech (1981)
13	Northcentral, Minnesota, USA	81	41	17	0	1980–1986	Fuller (1989)
14	Northeastern, Minnesota, USA	Not reported	23	0	7	1968–1974	Mech (1977)
15	Northeastern, Minnesota, USA	Not reported	7	2	0	1974–1976	Mech (1977)
16	Northwestern, Wyoming	299	142	17	0	1995–2004	Murray et al. (2010)
17	Central, Idaho, USA	219	90	31	0	1995–2004	Murray et al. (2010)
18	Upper Peninsula, Michigan, USA	367	178	68	0	1994–2013	O'Neil (2017)
19	Wisconsin, USA	1125	292	103	0	1979–2012	Treves et al. (2017b)
20	Southeastern, Yukon, Canada	78	25	0	0	1990–1993	Hayes and Harestad (2000)
21	Southeastern, British Columbia, Canada	14	3	0	0	2003–2006	Stotyn et al. (2007)
22	Northeastern, Alberta, Canada	18	3	0	1	1975–1978	Fuller and Keith (1980)
23	Westcentral, Alberta, Canada	33	7	0	2	2000–2001	Kuzyk et al. (2006)
24	Southwestern, Alberta, Canada	42	24	0	12	1987–2001	Callaghan (2002)
25	Southcentral, Ontario, Canada	26	17	0	5	1994–1998	Forshner et al. (2003)
Combined		3564	1442	329	225		

Note

Total represents all mortalities including harvest‐related and other caused mortalities. Data were retrieved from the CauseSpec database (Hill et al., 2019a) and a review of wolf literature. Mech (1977), Fritts & Mech (1981), and Murray et al. (2010) monitored multiple study sites which we treated as independent.

**TABLE 2:** Documented causes of wolf (*Canis lupus*) mortalities from 21 studies throughout North America (1968–2019)

Type	Cause	Total mortalities	No. used in legal harvest models
Anthropogenic	Legal harvest	225	225
	Illegal harvest	329	83
	Management related	174	36
	Vehicle collisions	96	10
	Train collisions	5	5
	Poisoning	4	4
	Other human‐related	40	24
Non‐anthropogenic	Disease	40	11
	Starvation	12	12
	Accident/ injury	7	6
	Predation	1	1
	Drowning	1	1
	Other natural	307	175
	Other animal‐related	77	30
	Unknown	124	51
Total		1442	674

Note

Mortality numbers across all studies reviewed are indicated by No. of total mortalities. For analyses of mortalities due to legal harvest, we excluded 7 studies; numbers in the final column represent this partial dataset.

### 3.2 Model results

**FIGURE 3:**
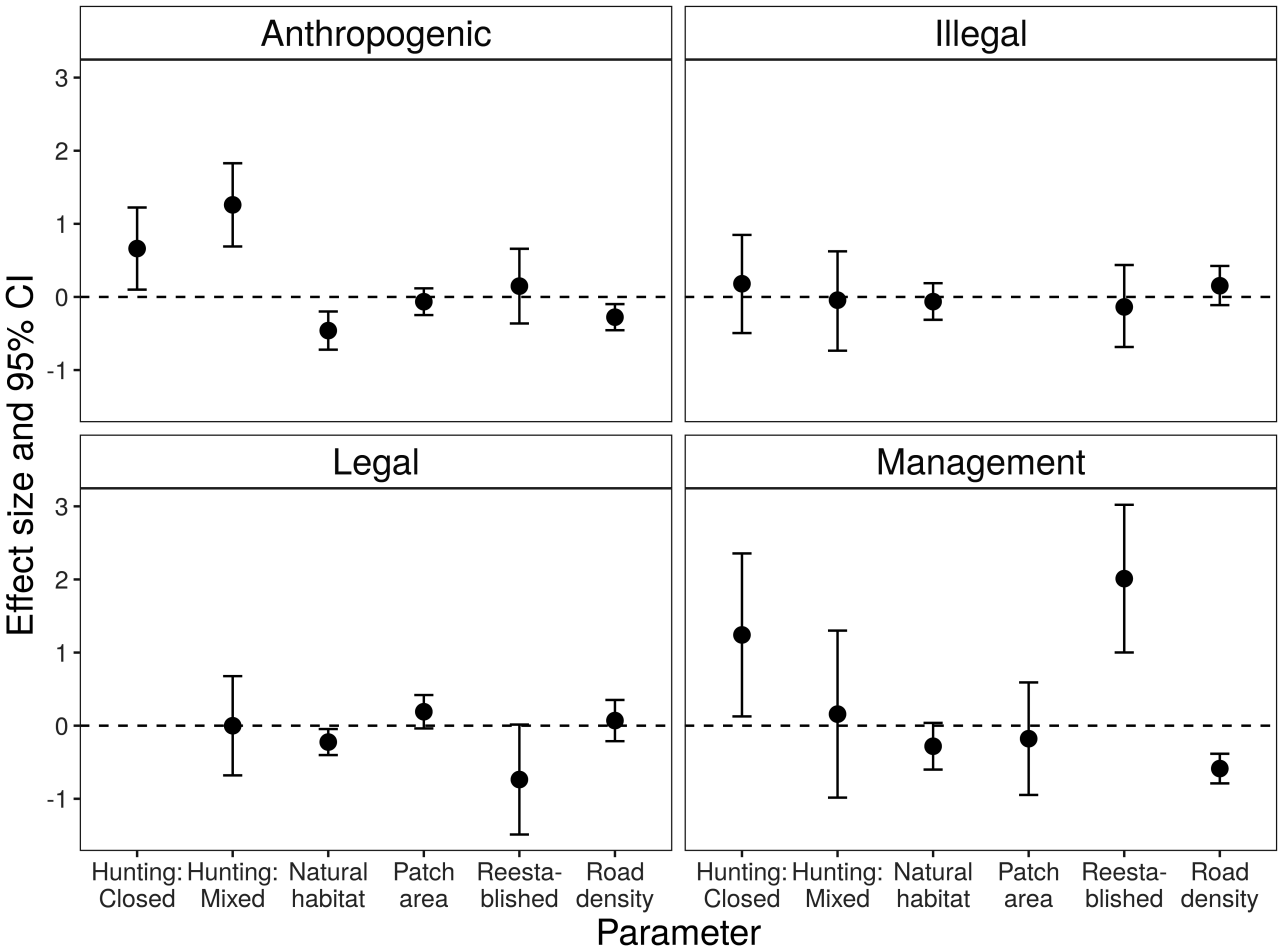
Covariate effect sizes on the proportion of wolf mortality in studies across North America during 1968–2019 due to all anthropogenic causes (*n* = 25 study sites), illegal harvest (*n* = 25), legal harvest (*n* = 17), and management action (*n* = 25). Covariates included hunting season status (closed and mixed, relative to a reference level of open), road density, mean patch area of natural habitat, total proportion natural habitat, and if the population had reestablished. Separate logistic regressions were fit for each mortality type, and continuous covariates were normalized before analysis. Error bars represent 95% confidence intervals (CI). We excluded the effect size for patch area for the illegal harvest model due to separation issues (see methods)

Model predictive accuracies as measured by AUC were 0.63 for the anthropogenic mortality model, 0.71 for the illegal harvest model, 0.61 for the legal harvest model, and 0.75 for the management removal model. The proportion of anthropogenic mortality was greater in areas with a closed hunting season and with mixed harvest regulations relative to areas with an open hunting season (odds of anthropogenic mortality 93% and 253% higher, respectively) (Figure 3, Table 3). The odds a wolf mortality was due to management removal were 246% greater if hunting was prohibited relative to regions with an open hunting season.

**TABLE 3:** Covariate effects on the proportion of wolf mortality in studies across North America during 1968–2019 due to all anthropogenic causes (*n* = 25 study sites), illegal harvest (*n* = 25), legal harvest (*n* = 17), and management action (*n* = 25)

	Anthropogenic		Illegal harvest		Legal harvest		Management	
Parameter	Log‐Odds	*p*	Log‐Odds	*p*	Log‐Odds	*p*	Log‐Odds	*p*
Intercept	0.17	.10	−6.15	<.01	−0.39	<.01	−3.86	<.01
Hunting season: Closed	0.66	.02	0.18	.60	N/A	N/A	1.24	.03
Mixed hunting regulations	1.26	<.01	−0.04	.90	0.00	.98	0.16	.79
Road density	−0.28	<.01	0.15	.26	0.07	.63	−0.59	<.01
Patch area mean	−0.07	.48	−22.84	<.01	0.19	.10	−0.18	.65
Natural habitat	−0.46	<.01	−0.06	.61	−0.22	.01	−0.28	.08
Reestablished	0.15	.57	−0.14	.63	−0.74	.06	2.01	<.01

Note

Covariates included hunting season status (closed and mixed, relative to a reference level of open), road density, mean patch area of natural habitat, total proportion of natural habitat, and if the study population had reestablished. Separate logistic regressions were fit for each mortality type, and continuous covariates were normalized before analysis. Due to separation issues with the patch area mean covariate, we re‐fit the illegal harvest model using Firth's penalized logistic regression.

The amount and configuration of natural habitat had similar effects on most mortality types. The amount of natural habitat was negatively correlated with the proportion of mortalities due to anthropogenic and legal harvest, with a similar but non‐significant pattern for management mortality. A one standard deviation increase in the proportion of natural habitat in the landscape (equivalent to a change of about 8 percent) reduced the odds of an anthropogenic mortality by 37% and legal harvest mortality by 20% (Figure 3, Table 3). The degree of fragmentation of natural habitat (i.e., lesser mean patch area) had a large negative effect on the proportion of mortality due to illegal harvest, but did not affect other mortality types. However, fitting the illegal harvest model with penalized likelihood did not completely resolve the separation issues observed with this covariate (Table 3), likely a consequence of no illegal harvest mortalities occurring at the 5 study sites with the greatest mean patch area mean.

Road density had a negative effect on the proportion of mortality due to anthropogenic and management sources but did not affect legal or illegal harvest mortality. A one standard deviation increase in road density (about 59 m/km^2^) decreased the odds of anthropogenic mortality by 24% and management mortality by 45% (Figure 3, Table 3). The proportion of mortalities due to management removals was significantly greater in reestablished wolf populations relative to permanent populations, but there was no difference for the other three mortality types.

## 4 DISCUSSION

Understanding how species are impacted by human activities is key for effective conservation and sustainable management of wildlife populations (Gantchoff et al., 2020). We did not find clear support for our prediction that wolf anthropogenic mortality would increase in areas with an open hunting season. We found that proportion of anthropogenic mortality was greater in areas where hunting was prohibited, and in areas with mixed hunting regulations. We found support for our prediction that the proportion of wolf management mortalities decreased when legal hunting was allowed, but found no support for illegal harvest increasing in areas where hunting was prohibited. Additionally, we found support for our prediction that wolf anthropogenic mortality would increase with lesser amounts of natural habitat, whereas our prediction of greater anthropogenic mortality at greater road densities was not supported.

The lack of clear influence of an open hunting season in total anthropogenic wolf mortality suggests that harvest mortality might not be fully additive nor compensatory. Other studies have shown divergent effects on wolf mortality from human‐induced causes. Implementation of a wolf hunting ban in Canada caused anthropogenic‐caused mortality to decrease, yet it was largely offset by natural mortality and wolf density remained relatively constant (Rutledge et al., 2010), indicating anthropogenic‐caused mortality was compensatory. In contrast, the combined effects of legal culling and disappearances (including verified poaching) halted wolf population growth in Europe (Liberg et al., 2020), suggesting their effects were at least partially additive. In Wisconsin (USA), anthropogenic‐caused mortality of wolves was mostly additive in early colonization and became partially compensatory as the population increased and expanded (Stenglein et al., 2018), suggesting small populations are more likely to be impacted by harvest. The absence of our expected relationship may have occurred because we could not account for wolf density, which can influence whether anthropogenic mortality is compensatory (Murray et al., 2010). Overall, for many North American large carnivores, it is unclear whether anthropogenic‐caused mortality sources are mostly compensatory or additive (Creel & Rotella, 2010; Gantchoff et al., 2020; Gude et al., 2012; Murray et al., 2010; Wolfe et al., 2015), and mortality is likely mediated by several population and landscape‐specific components.

Our results did not support a reduction in illegal harvest of wolves when a hunting season was authorized, unlike studies of wolves outside North America (Liberg et al., 2020). Alternatively, increased wolf poaching during periods of reduced protections (e.g., removal from U.S. Endangered Species Act) has been suggested (Santiago‐Avila, 2019). Illegal kills including poaching are difficult to quantify which hinders our understanding of mechanisms involved (Liberg et al., 2012). That an open hunting season or management removal decreases wolf poaching has long been debated, and both sides of the debate have supporters (Redpath et al., 2013; Woodroffe & Redpath, 2015) and critics (Chapron & Treves, 2016; Epstein, 2017). Although data limitations precluded a deeper analysis in our study, understanding the relative effects of legal versus illegal harvest on wolf mortality might be better evaluated (when data are available) by examining specific individuals removed from the population (e.g., adults vs. young, female vs. male, breeder vs. nonbreeder) to assess how disturbance to wolf social dynamics affects population structure and trajectory.

We found that mortality through management removal declined in study sites with an open hunting season vs. where it was prohibited. Unlike illegal harvest, management removal is legally authorized and readily quantified. As wolf density is closely associated with the prevalence of conflict with humans (Kompaniyets & Evans, 2017), reductions in wolf populations resulting from harvest may decrease the frequency of conflict and resulting mortality from management removal. Furthermore, some sources of human conflict increase with increasing pack size and smaller pack sizes following a hunting season could lead to reductions in such conflicts (Wydeven et al., 2004). Public harvest reduced livestock depredation by wolves in Montana, USA, provided a high enough proportion of the population was harvested (DeCesare et al., 2018). In addition, allowing local people to participate in wolf management removal (e.g., landowner permits for lethal take) may promote greater general acceptance of wolves because it enables people to manage conflicts on their own, increasing tolerance even if no open hunting season is implemented (Olson et al., 2019). Alternatively, our finding could be an artifact of the geographic distribution of study sites. Many of the study sites where legal harvests occurred (e.g., Alaska, USA) have low human population densities and few livestock. In contrast, sites where hunting was prohibited were in the southern portion of current gray wolf range with higher human populations and livestock densities. Livestock depredations by wolves are a primary cause for management removals (e.g., Ruid et al., 2009). Therefore, more frequent management removals in areas where hunting was prohibited could be more related to greater frequency of livestock and wolf depredations, than to hunting regulations.

In agreement with our predictions, increasing natural cover was associated with a decline in proportion of total human‐caused and legal harvest mortality. Areas of greater natural cover likely have less human use, reducing the likelihood of encounters between wolves and people (Barber‐Meyer et al., 2021). With greater cover, wolves may also have a more substantial prey base, reducing their reliance on anthropogenic food sources (Mohammadi et al., 2019). Similarly, wolves in Wisconsin were less likely to prey on livestock when there was greater forest cover (Treves et al., 2004). Anthropogenic mortality of brown bears and pumas also increased in locations with lesser extents of natural habitat (Moss et al., 2016; Wynn‐Grant et al., 2018). Additionally, natural cover fragmentation was associated with increased illegal harvest, suggesting that an increase in the human‐wildland interface results in increased risk of this mortality source. For many carnivores, anthropogenic mortality can be reduced by providing sufficient habitat that effectively segregates them from humans (Takahata et al., 2014). Our results indicate that maintaining large tracts of intact natural cover may reduce human‐caused mortality of wolves due to reduced human activity and consequently, potential for human‐wolf interactions.

Contrary to our predictions, higher road densities did not increase the likelihood of mortality from any anthropogenic source and were actually associated with lower proportions of overall anthropogenic and management mortality. Roads can serve as travel corridors for wolves (Hill et al., 2021) and the range of observed road densities and associated human activities may not have reached the threshold to facilitate greater anthropogenic mortality. Higher road densities generally correspond with more developed areas and decreased prevalence of human‐wildlife conflict, including lower probability of wolf‐livestock depredations across some of the regions included in our analysis (e.g., Fowler et al., 2019). In contrast to our results, wolf legal harvest increased with road density in other areas, likely because roads act as points of access (Person & Russell, 2008). The effects of roads are likely context‐dependent, being stronger if overall road densities are low (i.e., remote areas). The effect of roads on wolf mortality may also be dependent on spatial scale, with higher mortality near roads, but not overall across the study area. Moreover, wolves’ use of roads is complex as they appear to use roads for ease of travel while employing avoidance behavior toward humans (Bojarska et al., 2020; Hill et al., 2021; Kautz et al., 2021; Zimmermann et al., 2014).

In agreement with our predictions, reestablished populations experienced greater proportions of mortalities from management removal. However, other mortality sources were not influenced by whether wolves had reestablished. Carnivore reintroduction may increase livestock depredations, but effective livestock management overtime can reduce such negative interactions (Strand et al., 2019). Furthermore, longer periods of coexistence with carnivores also leads to increased tolerance by humans, likely reducing the frequency of landowner complaints (Zimmermann et al., 2001). Therefore, as wolf populations become reestablished more effective livestock management and more favorable opinions of them may lead to reduced management removal. Additionally, reintroduced populations were those from the southern portion of the study region. The northern study sites have less dense human populations, and wolves in these areas are more likely to be harvested for use by indigenous communities (Fritts et al., 2003). As a result, recolonization status may be correlated with other study area attributes that influence cause‐specific mortality.

Inferences derived by combining studies conducted across such large geographic and temporal scales are inherently subject to biases. For example, we were unable to evaluate site‐specific attributes such as local attitudes toward wolves or local management strategies that may influence mortality estimates (Treves et al., 2013). Wolf density may influence mortality patterns (Murray et al., 2010), and although we incorporated reestablishment status which could serve as a coarse proxy for wolf density, we could not account for wolf density. Additionally, we excluded mortalities of unknown cause that represented 8.6% of total mortalities and may be biased toward certain mortality sources such as illegal harvest (Treves et al., 2017). Therefore, our conclusions regarding some mortality sources may have been underestimated compared with the other mortality sources we examined. Lastly, we were unable to calculate cause‐specific mortality rates due to data availability limitations, which could produce different results compared to analysis of proportional mortality.

Persistence of top mammalian predators in expanding human‐modified landscapes is a major conservation challenge (Lamb et al., 2020). In North America, areas with open wolf hunting seasons had lower management removals but not lower illegal harvest, and did not result in a pronounced increase in total anthropogenic mortality. Different types of human‐caused mortality can impact not only wolf abundance, but also social patterns, suggesting that conservation planning should consider the potential effects of harvest regulations beyond population sizes (Bassing et al., 2020; Rutledge et al., 2010). Moreover, although carnivores can adapt to humans, there is a strong need to understand and integrate their ecology and management in human‐modified landscapes (Carter & Linnell, 2016). In particular, our observed association between large patches of natural habitat and reductions in several sources of human‐caused wolf mortality reiterate the importance of habitat preservation to maintain wolf populations (e.g., Barber‐Meyer et al., 2021). Furthermore, increased positive attitudes toward wolves and other large carnivores benefits species’ persistence (e.g., Gompper et al., 2015). Effective conservation and management of wide‐ranging carnivores, particularly species with expanding ranges, will depend on multifaceted strategies that integrate ecological and socioeconomic factors to facilitate their long‐term coexistence with humans.

## CONFLICT OF INTEREST

The authors declare no competing interests.

## AUTHOR CONTRIBUTIONS

**Jacob E. Hill:** Conceptualization (equal); Investigation (equal); Methodology (equal); Supervision (equal); Writing – original draft (lead); Writing – review & editing (lead). **Hailey M. Boone:** Conceptualization (equal); Investigation (equal); Methodology (equal); Visualization (lead); Writing – review & editing (equal). **Mariela G. Gantchoff:** Conceptualization (equal); Investigation (equal); Methodology (equal); Supervision (equal); Writing – original draft (lead); Writing – review & editing (lead). **Todd M. Kautz:** Conceptualization (equal); Data curation (equal); Investigation (equal); Methodology (equal); Writing – review & editing (equal). **Kenneth F. Kellner:** Conceptualization (equal); Data curation (equal); Formal analysis (lead); Investigation (equal); Methodology (equal); Visualization (lead); Writing – original draft (equal); Writing – review & editing (equal). **Elizabeth K. Orning:** Conceptualization (equal); Investigation (equal); Methodology (equal); Writing – review & editing (equal). **Jamshid Parchizadeh:** Conceptualization (equal); Investigation (equal); Methodology (equal); Writing – review & editing (equal). **Tyler R. Petroelje:** Conceptualization (equal); Data curation (equal); Investigation (equal); Methodology (equal); Writing – review & editing (equal). **Nathaniel H. Wehr:** Conceptualization (equal); Data curation (lead); Investigation (equal); Methodology (equal); Writing – original draft (equal); Writing – review & editing (equal). **Shannon P. Finnegan:** Conceptualization (equal); Methodology (equal); Writing – review & editing (equal). **Nicholas L. Fowler:** Conceptualization (equal); Methodology (equal); Writing – review & editing (equal). **Ashley L. Lutto:** Conceptualization (equal); Methodology (equal); Writing – review & editing (equal). **Alejandra Zubiria Perez:** Conceptualization (equal); Methodology (equal); Writing – review & editing (equal). **Sarah L. Schooler:** Conceptualization (equal); Methodology (equal); Writing – review & editing (equal). **Merijn van den Bosch:** Conceptualization (equal); Methodology (equal); Writing – review & editing (equal). **Jerrold L. Belant:** Conceptualization (equal); Funding acquisition (lead); Methodology (equal); Resources (lead); Supervision (equal); Writing – review & editing (equal).

## Supporting information

Supplementary Material

## Data Availability

All data and associated references are included in the Supplementary Information.
